# Type 2 Diabetes and Risk Factors in an Adult Population in Oujda, Eastern Morocco: A Cross-Sectional Observational Study

**DOI:** 10.7759/cureus.65715

**Published:** 2024-07-29

**Authors:** Abdellatif Maamri, Souad Ben El Mostafa, Dirk Vissers, Bart Van Rompaey

**Affiliations:** 1 Department of Biology, Faculty of Sciences, Université Mohammed Premier, Oujda, MAR; 2 Department of Rehabilitation Science and Physiotherapy, Faculty of Medicine and Health Sciences, University of Antwerp, Antwerp, BEL; 3 Department of Family Medicine and Population Health, Faculty of Medicine and Health Sciences, University of Antwerp, Antwerp, BEL

**Keywords:** health centers, women, risk factors, morocco, diabetes

## Abstract

Objectives

The rapidly increasing prevalence of diabetes makes it a public health concern. Adopting a healthier lifestyle can prevent or delay the onset of type 2 diabetes (T2D), the most common type of diabetes, and its complications. The aim of this study is to determine the prevalence of diabetes at the level of local health centers in the prefecture of Oujda, Morocco, and its relationship with obesity, physical activity, and sociodemographic factors.

Methodology

In a cross-sectional study in first-line health centers, sociodemographic and bioclinical data were collected through convenient purposive sampling using anthropometric and blood glucose measurements and structured, comprehensible interview questionnaires. The International Physical Activity Questionnaire (IPAQ) questionnaire was used to measure the physical activity of the patients. The association of T2D with age, gender, education, occupation, obesity, and physical activity was analyzed.

Results

Out of 535 observed patients, 510 were included, mostly female with a minimum age of 18 years, with a prevalence of T2D of 16%. More than half of the patients were illiterate (56%) and 83% had no occupation. Obesity was prevalent, especially among diabetics, and only a minority were physically active according to WHO targets. Thirty-six percent of all patients and 46% of diabetics reported low levels of physical activity. Age and obesity were the main factors associated with T2D.

Conclusion

Obesity and T2D have a high prevalence in the Oujda region. A balanced diet and regular physical activity remain our best recommendations for preventing this disease. Special attention should be paid to women with diabetes in Arab countries so that they can actively participate in prevention activities.

## Introduction

Diabetes is a common metabolic disorder, and its devastating effect is increasing alarmingly every day reaching epidemic proportions [1]. This pathology is characterized by chronic hyperglycemia due to dysregulated carbohydrate metabolism, which also affects proteins and fats. The main cause is total or relative insufficiency of insulin action [2]. According to the 2021 estimates from the World Health Organization (WHO) and the International Diabetes Federation (IDF), 422 million people worldwide suffer from diabetes. This number is expected to increase to 552 million by 2030. Moreover, one person in ten has diabetes, and half of those with the disease remain undiagnosed [3]. The global prevalence of diabetes was 382 million people in 2013, with a projection of 592 million people by 2035, according to a 2019 report [4]. Three types of diabetes are described: type 1 diabetes, type 2 diabetes (T2D) and gestational diabetes. Pathophysiologically, T2D is due to alterations in the β-cell function associated with insulin resistance being present for many years before the onset of hyperglycemia [5]. In particular, T2D seems to have the highest increase in prevalence. This type of diabetes represents 90% of all diabetes and is largely the result of overweight and a sedentary lifestyle, with 80% of the affected patients being overweight or obese [6]. The main risk factors for the persistent wave of T2D are urbanization, lifestyle changes, and especially physical inactivity and obesity, which threaten people every day [7,8]. T2D, which has been silent for many years, represents a major public health problem due to its current progression.

Arab countries are not immune to this pandemic. By cluster type, the highest prevalence was observed in Gulf Cooperation Council (GCC) countries with 25.5%, while non-GCC countries had the lowest prevalence (12.7%). The combined mean prevalence of T2D in Arab GCC and non-GCC countries was 16.2% [9]. According to the IDF Atlas 2021, the Middle East and North Africa (MENA) region has the highest regional prevalence with 73 million adults living with diabetes. This could increase to 136 million by 2045. It was also reported to have the highest proportion of deaths from diabetes in people of working age in 2021 (24.5%) [3,10]. In Morocco, more than two million people over the age of 25 have diabetes; 50% of all diabetics remain undiagnosed and unaware of their condition [11]. The Ministry of Health in Morocco showed a significant increase in the number of adult diabetics evolving from 1.5 million in 2011 to more than 2 million in 2015. T2D accounts for 80% of diabetes in Morocco. Diabetes is mainly related to obesity and lifestyle, including physical activity and diet. T2D directly causes more than 12,000 deaths per year in Morocco and leads to an additional 32,000 deaths. These are attributable to complications resulting from high blood glucose levels and are generally the result of an unbalanced diet and lack of physical activity, leading to overweight and obesity. At the economic level, the management of diabetes costs the State of Morocco 11 billion dirhams ($ 2,994,806,650 or € 2.837,999,094 on 10/06/2023) at a time when the healthcare budget does not exceed 14 billion dirhams per year [11-13].

The main objective of this article is to explore the prevalence of T2D and its relationship with physical activity, obesity, and socio-demographic factors in primary healthcare center attendees in the Oujda prefecture in oriental Morocco.

## Materials and methods

This is a descriptive and analytical cross-sectional epidemiological study that collected data on physical activity, obesity, and other variables related to T2D. Respondents were included for opportunistic screening between February and August 2019 during a regular visit to one of the 33 health centers in Oujda. The latter are first-line health structures that mainly provide primary care and stimulate public health by organizing prevention, education, and health promotion activities.

Patients were included once if they were randomly attending the health center at the time of the study. They had to be 18 years of age or older, both male and female, without prior selection for complaint or pathology. No other inclusion or exclusion criteria were used. This was a non-probability convenience sample conducted with a maximum of 40 patients at each health center, with each center enrolling patients according to its staffing or structural capabilities. In this cross-sectional study, a sample of 500 patients was calculated to be sufficient for the analysis of risk factors.

The variables were collected using the following instruments:

Anthropometric measurements

Height was measured in centimeters using a mobile height gauge. Bodyweight was measured using the impedance meter Omron BF-511 (OMRON Healthcare Europe B.V., Nogent-sur-Marne, France). This device calculated the body mass index (BMI), which allows the weight status to be classified according to WHO guidelines: lean (BMI lower than18.5), normal weight (BMI from 18.5 to 24.9), class I obesity - overweight (BMI from 25 to 29.9), class II obesity - obese (BMI from 30 to 39.9) and class III - morbid obesity (more than 40).

Blood glucose level

Blood glucose levels in capillary blood from the finger were measured using the On Call Plus II Monitoring System (Acon Laboratories Inc., San Diego, California, USA). Except for already known diabetic patients, diabetes was diagnosed when fasting blood glucose was >1.26 g/dL, or blood glucose at least two hours after the last meal was >2 g/dL. A subsequent medical consultation was scheduled to confirm or rule out diabetes. Known diabetics were also tested for HbA1c. The diagnosis of T2D was then made by the attending physician.

Questionnaire for diabetes and obesity screening

The diabetes and obesity screening questionnaire was developed in Morocco in a different population [14]. There, the included subjects participated in interviews conducted by trained interviewers. These questionnaires were targeted to all selected individuals, using simple and understandable sentences and avoiding terms that could cause ambiguity.

International Physical Activity Questionnaire

Physical activity was assessed using the short version of the self-report International Physical Activity Questionnaire (IPAQ). This open access questionnaire is publicly available without cost or permission. The IPAQ calculates the amount of Metabolic Equivalents of Task (METs) where one MET is the amount of energy expended by a person at rest. The calculation of METs allows the classification of physical activity into three levels (IPAQ categories): high, moderate, and low [15].

Statistical analysis

Statistical analysis was performed using IBM SPSS Statistics for Windows, Version 29 (Released 2023; IBM Corp., Armonk, New York, United States). Descriptive statistics were used to analyze population characteristics. Distributions of continuous data were tested for normality using the Kolmogorov-Smirnov test. Continuous variables were analyzed using the independent t-test. Chi-squared and Fisher exact tests were used to compare categorical variables. The association with the presence or absence of T2D was tested with binary logistic regression, with odds ratios (exp.(B)) and 95% confidence intervals. To calculate the multivariate model, variables were entered separately by magnitude of exp(B) if they improved the model. A p-value less than 0.05 was considered significant.

Statements and declarations

The principles of good research and ethical standards protected all subjects involved in the study. These principles consist of protecting the subjects while ensuring confidentiality and respecting their privacy. The study was approved by the Ethics Committee of the Regional Health Directorate in Oujda (EC 128/18). Written informed consent was obtained from each participant prior to the implementation of any protocol. To make a well-considered decision, they were informed about the aspects of the study and its objectives.

Participation was completely voluntary; all respondents were free to accept or decline to participate in the study, with no consequences for their further treatment. Once enrolled, patients had the right to withdraw from the study at any time without prejudice.

## Results

Participants’ characteristics

Of the 535 patients' data files collected, 510 were sufficiently complete to be included in the study. Women (86%) made up the majority of the study population. The mean age of the patients was 46.4 years (±15.2 years). More than half of them were illiterate and had never attended school, one quarter of them had attended only a Koranic or primary school. The rest had at least a secondary education including a minority with a higher education degree. The vast majority (86%) were unemployed or retired (Table 1). According to the BMI results, one-quarter of the study population had a normal body weight, and 71% were overweight. Based on the data from the IPAQ questionnaire, 43% were highly physically active, 21% had a moderate level of physical activity, while the remaining 36% had a low level of physical activity (Table 1).

**Table 1:** Description of the included population Differences were calculated using the independent t-test (*) for continuous variables, chi-squared or Fischer exact ($) for categorical variables T2D: Type 2 diabetes

				Negative for T2D		Positive for T2D		
				84%	n=428	16%	n=82	
Continuous variable descriptive			Mean (SD)	Mean	(SD)	Mean	(SD)	p
Age (years)	(n=503)		46.64 (15.2)	44.7	14.8	56.8	12.9	<0.001^*^
BMI (kg/m²)	(n=510)		28.7 (SD 6.3)	28.2	6.3	31.5	5.4	<0.001^*^
Categorical variables descriptive			n	%	n	%	n	p
Gender	(n=509)	Female	436	84.6%	369	15.4%	67	0.17^$^
		Male	73	79.5%	58	20.5%	15	
highest education	(n=494)	No education	278	82.4%	229	17.6%	49	0.07^$^
		Primary or Koranic school	128	85.2%	109	14.8%	19	
		Secondary school	65	93.8%	61	6.2%	4	
		Higher education	23	73.9%	17	26.1%	6	
profession	(n=510)	No profession	422	84.6%	357	15.4%	65	0.05^$^
		Worker	11	72.7%	8	27.3%	3	
		Public sector employee	9	44.4%	4	55.6%	5	
		Private sector employee	5	100.0%	5	0.0%	0	
		Liberal profession	18	83.3%	15	16.7%	3	
		Student	7	85.7%	6	14.3%	1	
		Other	24	91.7%	22	8.3%	2	
		Retired	14	78.6%	11	21.4%	3	
WHO class of obesity	(n=510)	Underweight	18	100.0%	18	0.0%	0	<0.001^$^
		Normal	128	93.8%	120	6.3%	8	
		Class I obesity - overweight	157	82.8%	130	17.2%	27	
		Class II obesity - obesity	183	77.0%	141	23.0%	42	
		Class III obesity - extreme obesity	24	79.2%	19	20.8%	5	
physical activity	(n=505)	Low	180	80.6%	145	19.4%	35	0.19^$^
		Moderate	107	85.0%	91	15.0%	16	
		High	218	87.2%	190	12.8%	28	

Prevalence of T2D

The prevalence of diabetes was 16.1%, of which one-quarter had never been diagnosed. The age of the diabetic patients ranged from 18 to 89 years (mean 56.8, ±13 years). Age, BMI, and different types of obesity were significantly associated with a positive diagnosis of T2D. Occupation, educational level, and physical activity level were not, although some trends were observed (Table 1). Low physical activity and obesity were most common in diabetic patients (Table 2).

**Table 2:** Activity (IPAQ category) and obesity in individuals being positive for diabetes Results were complete for n=59 diabetic patients, and the p-value for difference between categories was calculated with the chi-squared test IPAQ: International Physical Activity Questionnaire

p-value chi-squared=0.33		Activity (IPAQ category)		
		Low	Moderate	High
Obesity	Not obese (n=5)	40% (n=2)	0% (n=0)	60% (n=3)
	Obese (n=54)	46% (n=25)	22% (n=12)	32% (n=17)
Total %		46% (n=27)	20% (n=12)	34% (n=20)

Binary logistic regression confirmed that age and obesity were predominant factors in the development of T2D. In the multivariate regression model, variables were entered if they improved the model with diabetes as the outcome. Variables representing similar observations were not used together in the multivariate model, e.g., BMI and obesity. The variable with the largest OR after univariate analysis was selected. The final model retained 485 cases in the analyses and showed a Nagelkerke R² of 22.4%. After multivariate analysis, again age and obesity remained the predominant factors influencing being positive for T2D. The educational level and physical activity improved the model without being statistically significant (Table 3).

**Table 3:** Univariate and multivariate binary regression with being positive for diabetes as the outcome Multivariate model fit of 22.3% for n=485 in analysis, Nagelkerke R² was used for the calculation of associations and model fit OR: Odds ratio; CI: confidence interval; adj: adjusted

Variables		Univariate binary regression					Multivariate binary regression			
		OR (exp (B))	p	CI 95 % low	CI 95 % high	Nagelkerke R² (univariate)	adj OR (exp (B))	p	CI 95 % low	CI 95 % high
Age		1.06	0.000	1.04	1.078	0.144	1.07	0.000	1.04	1.09
Highest education	No education	ref	ref	ref	ref	0.028	ref	ref	ref	ref
	Primary or Koranic school	0.82	0.49	0.46	1.45		1.2	0.64	0.6	2.3
	Secondary school	0.31	0.03	0.11	0.88		0.7	0.55	0.2	2.2
	Higher education	1.65	0.32	0.62	4.4		4.4	0.01	1.3	14.1
Obesity binomial	Not obese	ref	ref	ref	ref	0.066	ref	ref	ref	ref
	Obese	4.4	0.000	2.07	9.39		5.59	0	2.14	14.62
Physical activity	Low	ref	ref	ref	ref	0.011	ref	ref	ref	ref
	Moderate	0.73	0.34	0.38	1.39		1.18	0.66	0.56	2.48
	High	0.61	0.07	0.36	1.05		1.03	0.92	0.54	1.97

## Discussion

The present study was carried out in first-line public health centers of Oujda. This is a predominantly urban area, with only six rural health centers among the 33 that we studied. The aim was to determine the association between T2D, obesity, and physical activity. Age and obesity were associated and most of the diabetic patients had low levels of physical activity. The high rate of women is due to their more frequent visits to the local health centers. The purpose of these visits is to monitor chronic diseases such as diabetes and hypertension, to take their children to the doctor’s visit, or to have babies vaccinated.

A higher than national prevalence of T2D was reported in this population. While 6.6% of the population had diabetes in 2000, this number increased dramatically in Morocco between 2000 and 2017, more in women than in men. In Oujda, the prevalence was 10% in 2018, at that time 2.4% lower than the national level [7,11]. In Algeria, a similar prevalence of 10.4% was observed in the region of Tlemcen, adjacent to Oujda [16]. The higher prevalence compared to previously reported figures may indicate a more prolonged rise in the T2D pandemic or a higher prevalence in the lower strata of the population.

Although increasing age was significantly associated with the presence of T2D in our study, half of the diabetic patients included were younger than 57 years. In the study by Gharbi et al. (2016), two-thirds of the subjects were older than 40 years [17]. While this confirms that T2D manifests itself with age, younger age groups are increasingly at risk. As they do not use health services as frequently as older people, a wide range of screening may be needed in the younger adult population to detect the early onset and the overall prevalence of T2D.

Other studies in the cities of Oujda and Marrakech showed that women were more affected than men. One of the reasons may be that Arab women in general tend to be more sedentary and do not exercise indoors or in public. Therefore, there seems to be a gender difference in the incidence of diabetes with a female predominance [18,19]. In our study, this association was not significant, probably because the majority of patients were female. However, specific preventive measures for female diabetics are also advisable here, since women are the largest gender group attending the local health centers.

Urbanization remains a discriminating parameter in the spatial disparities of diabetes [20]. Our results are predominantly from centers in urbanized communities. Westernizing urban lifestyles lead to more unhealthy eating patterns and less physical activity. In Tlemcen, the higher prevalence of T2D in urban areas was related to the region's difficult economic situation and the social transitions that the country underwent at the end of the 20th and the beginning of the 21st century. This has stimulated phenomena of stress and moral instability that favor the onset of metabolic diseases, coupled with an increase in the prevalence of obesity and diabetes [16]. In Morocco, this nutritional transition, which has affected the Oriental region since the year 2000, has already been described [21].

A large proportion of our sample was illiterate and unemployed. The prevalence of diabetes seems to be higher in this part of the population. Therefore, socio-demographic characteristics seem to have an impact on the presence of T2D, the follow-up of recommended examinations, and the occurrence of its complications as well as on the adapted diet [22]. The development of tailored interventions for T2D must consider the illiteracy and low education of the majority of the population in local health centers. 

Obesity increases the risk of diabetes by a factor of ten in men and by a factor of eight in women [23]. Our results confirmed that T2D and obesity are associated. This is consistent with other studies showing that the risk of developing T2D rises sharply with body mass index and that weight loss is associated with a reduction in the risk of diabetes, especially in overweight and obese individuals [24]. Almost all the diabetic patients included were overweight or obese. The WHO states that obesity is more prevalent in the least privileged categories than in the most affluent [25]. According to the ObEpi study (national epidemiological survey on overweight and obesity 2012), the obesity rate is higher among people with a primary school education than among those with a postgraduate degree [26]. Another study by the Research Center for the Study and Observation of Living Conditions (Centre de recherche pour l ‘étude et l ‘observation des conditions de vie, Crédoc) in France showed that the most educated people have the healthiest eating habits (more fruit and vegetables, higher nutrient intake, better nutritional indices, etc.). This is because they are the most interested in knowing how food affects health [27]. In Morocco, however, people live in a society that often believes that being overweight or obese is a sign of good health and wealth.

Almost half of the diabetics in our study reported low physical activity, although this was not statistically significantly different from others. Nevertheless, this trend supports the role of the association with T2D. Regular physical activity delays the onset of diabetes in individuals at risk for T2D and reduces the risk of cardiovascular morbidity and other complications [28]. According to the WHO in 2016, 28% of adults aged 18 years and older were insufficiently physically active, defined as less than 150 minutes of moderate or vigorous physical activity per week [29]. The overall prevalence of physical inactivity was 32.3%. The prevalence among men and women was 28.8% and 35.5%, respectively. The prevalence among non-Arabs and Arabs was 28.6% and 43.7%, respectively. Women and Arabs were more likely to be physically inactive than their counterparts. In a US study of 556 people in 38 Arab countries, the prevalence of physical inactivity was higher in the Muslim world than in non-Muslim countries [30]. Lack of awareness of the benefits of physical activity, lack of adapted sports facilities for women, and financial inaccessibility are likely to be the main reasons for this situation.

The strengths of this study lie in several areas. First, this is an independent screening study in primary care public health centers without regard to patients' known medical history. Previously published data are from a more clinical setting. In addition, the prevalence was observed in a lower stratum of Moroccan society. The broad screening in different centers gives a picture of the prevalence of T2D in this specific population. The first limitation is that we started with opportunistic screening of people who presented with health complaints. Thus, the prevalence might be different if a general population-based study with stratified samples was conducted. On the other hand, our results may not reflect the situation of the general Moroccan population, since we studied specific patients in the largest city in the eastern part of Morocco. In addition, the use of the self-reported IPAQ questionnaire has its limitations. A more objective approach to assessing activity could add important information.

This study calls for interventions for T2D in local health centers that are tailored to women who are not employed and have a lower level of education. Promotion of physical activity and education on healthy eating habits are important contributions in the fight against the T2D pandemic.

## Conclusions

The prevalence of T2D in a sample of patients attending local health centers in eastern Morocco was 16%. T2D's association with age and obesity was confirmed in this population. Trends for association with low physical activity and socioeconomic status were also noted. An alarming number of young adults develop diabetes. Women attending local health centers need to be targeted for physical activity and nutrition education.
